# The Stability-Indicating Ultra High-Performance Liquid Chromatography with Diode Array Detector and Tandem Mass Spectrometry Method Applied for the Forced Degradation Study of Ritlecitinib: An Appraisal of Green and Blue Metrics

**DOI:** 10.3390/ph18010124

**Published:** 2025-01-17

**Authors:** Jelena Kovačić, Daniela Amidžić Klarić, Nikša Turk, Željko Krznarić, Emma Riordan, Ana Mornar

**Affiliations:** 1 Department of Pharmaceutical Analysis, Faculty of Pharmacy and Biochemistry, University of Zagreb, 10000 Zagreb, Croatia; jelena.kovacic@pharma.unizg.hr (J.K.); daniela.amidzic@pharma.unizg.hr (D.A.K.); 2 Department of Gastroenterology, University Hospital Centre, 10000 Zagreb, Croatia; niksa.turk@gmail.com (N.T.); zeljko.krznaric60@gmail.com (Ž.K.); 3 School of Medicine, University of Zagreb, 10000 Zagreb, Croatia; 4 School of Pharmacy, University College Cork, T12 YT20 Cork, Ireland; 121470174@umail.ucc.ie

**Keywords:** ritlecitinib, JAK3 inhibitor, stability-indicating method, forced degradation study, green analytical chemistry, degradation kinetics, HPLC, mass spectrometry

## Abstract

Background/Objectives: Janus kinase inhibitors open new horizons for small-molecule drugs in treating inflammatory bowel disease, with ritlecitinib demonstrating significant efficacy in clinical trials for ulcerative colitis and Crohn’s disease. Ritlecitinib, a second-generation JAK3 inhibitor, is a novel therapeutic agent for alopecia areata and other autoimmune conditions. Methods: A new stability-indicating UHPLC-DAD-MS/MS method was developed, validated, and applied for a forced degradation study of ritlecitinib under ICH guidelines. Results: The method demonstrated high specificity, sensitivity (LOD: 0.04 µg/mL; LOQ: 0.14 µg/mL), precision (RSD ≤ 0.15%), and accuracy (99.9–100.3%). Forced degradation studies under acidic, basic, oxidative, thermal, and photolytic conditions revealed four novel degradation products. Basic degradation followed second-order kinetics, while oxidative degradation followed zero-order kinetics. Conclusions: The validated method reliably characterized ritlecitinib’s stability and degradation products, providing essential data for optimizing formulation, determining proper storage conditions, anticipating drug–excipient interactions, and ensuring quality control. The eco-friendliness and applicability of the developed forced degradation procedure were evaluated using various green and blue metric tools. Incorporating green analytical principles underscores its potential for sustainable pharmaceutical analysis.

## 1. Introduction

Janus kinases (JAKs) are intracellular, multidomain non-receptor tyrosine kinases that play pivotal roles in the transduction of cytokine-mediated signals. The JAK family consists of four members (JAK1, JAK2, JAK3, and tyrosine kinase—TYK2) that relay signals downstream of type I and type II cytokine receptors through signal transducers and activators of transcription (STATs). The JAK-STAT signaling pathway, as an evolutionarily conserved pathway, is crucial in innate immunity, adaptive immunity, hematopoiesis, and disorders that frequently appear in cancer, autoimmune, and inflammatory disorders [1].

Based on this, the targeting of JAK-associated pathways to prevent its phosphorylation using JAK inhibitors has rapidly entered the clinical arena for treating a wide array of inflammatory, autoimmune, and myeloproliferative diseases, improving many classic therapeutic algorithms. As of now, 13 JAK inhibitors, typically administered once or twice daily in oral (tablet, capsule, and solution) and topical (cream) formulations, have been approved by relevant regulatory authorities [2]. Despite their increasing popularity, significant discussions regarding their safety profiles have arisen. First-generation JAK inhibitors, which act in an orthosteric manner by occupying the phosphotransferase pocket of JAKs, inhibit multiple JAKs and interfere with the biological activity of many factors. The side effects of first-generation JAK inhibitors can be partially attributed to their lack of selectivity, prompting recent research to focus on developing second-generation highly selective JAK inhibitors aimed at increasing therapy precision and reducing off-target activity [3,4].

**Figure 1:**
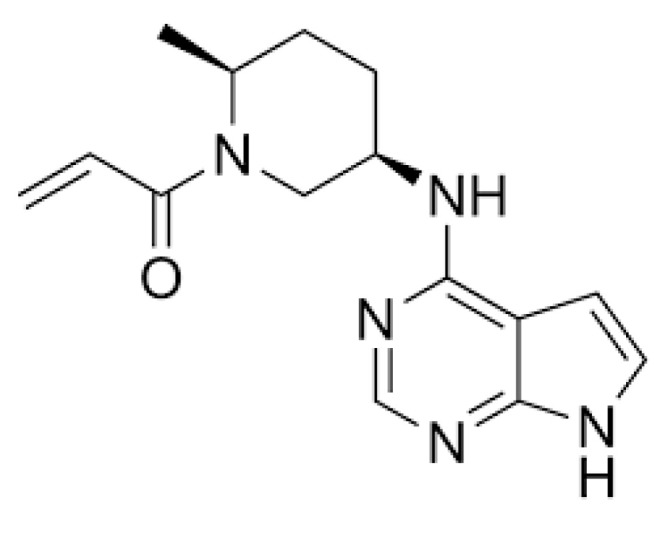
Structure of the irreversible Janus kinase 3 and tyrosine kinase inhibitor, ritlecitinib.

Ritlecitinib (1-[(2S,5R)-2-methyl-5-(7H-pyrrolo[2,3-d]pyrimidin-4-ylamino)piperidin-1-yl]prop-2-en-1-one; WHO ACT code L04A-F08; CAS No: 1792180-81-4; PF-06651600) is an irreversible inhibitor of JAK3 and TYK2 that Pfizer is developing under the trade name LITFULO^TM^ for treatment of alopecia areata (Figure 1).

This new therapeutic solution for severe alopecia areata was first approved in the USA on 23 June 2023, followed by Japan on 26 June 2023. It received a marketing authorization valid throughout the European Union, including Iceland, Liechtenstein, and Norway on 15 September 2023. Thereafter, the UK Medicines and Healthcare Products Regulatory Agency authorized LITFULO^TM^ on 1 November 2023. It became the first approved treatment for severe alopecia areata in Canada on 13 February 2024 [5].

Moreover, ritlecitinib, as a highly selective and orally bioavailable JAK3 inhibitor, represents a potential immunomodulatory therapy. Due to its favorable efficiency, safety, tolerability, and bioavailability, it has been under clinical studies for the treatment of other conditions. Randomized, double-blind, multicenter, phase 3 trials (ClinicalTrials.gov identifier: NCT05583526—TRANQUILO, and ClinicalTrials.gov identifier: NCT06072183—TRANQUILO 2) are currently recruiting patients to investigate the efficacy and safety of ritlecitinib in patients with vitiligo. JAK inhibitors are opening up new horizons for small molecule drugs in inflammatory bowel disease treatment strategies. Thus, the efficiency of ritlecitinib in treating moderate to severe active ulcerative colitis was reported in the VIBRATO phase 2b study (ClinicalTrials.gov identifier: NCT02958865). Furthermore, ritlecitinib met the primary endpoint during the induction period, demonstrating significant clinical improvements versus placebo in patients with active Crohn’s disease (ClinicalTrials.gov identifier: NCT02958865—PIZZICATO).

From the stages of drug research and development to marketing and post-marketing, analytical techniques play a vital role in the pharmaceutical industry in ensuring quality control, regulatory compliance, process optimization, troubleshooting, and batch release. As drug development progresses, analytical methods are refined and expanded, based on enhanced knowledge of the active pharmaceutical ingredient and drug product, as well as the requirements specific to each development and clinical application stage. Finally, analytical methods are included in the compendial monographs of relevant pharmacopeias to characterize the quality of bulk drug materials and pharmaceutical products. The main objective of a stability-indicating method is to monitor results during stability studies to guarantee safety, efficacy, and quality. These methods help to ensure that the final product meets the required specifications, complies with regulatory requirements, and provides safe and effective patient treatment [6,7,8,9].

The literature on liquid chromatographic techniques for the determination of ritlecitinib in pharmaceutical and biomedical samples is limited. There are only two studies about the determination of ritlecitinib concentration levels in rat and human biofluids [10,11]. To the best of our knowledge, forced degradation studies and development of stability-indicating analytical methods were not reported on ritlecitinib. Hence, to predict the stability of ritlecitinib, there is a tremendous need to carry out a comprehensive study of the forced degradation of the drug substance under the various forced degradation conditions conducted by the International Council for Harmonization of Technical Requirements for Pharmaceuticals for Human Use (ICH) guidelines.

Herein, we report (i) a validated stability-indicating method using ultrahigh-performance liquid chromatography coupled with a diode-array detector and tandem mass spectrometer (UHPLC-DAD-MS/MS), which successfully separated four degradation products; (ii) a comprehensive forced degradation study of ritlecitinib under hydrolytic, oxidative, thermal, and photolytic conditions as prescribed by ICH guidelines; (iii) an identification of degradation products; (iv) a discussion on degradation kinetics of ritlecitinib; and (v) an evaluation of the ecological character of the proposed analytical procedure.

## 2. Results and Discussion

### 2.1. Method Development and Optimization

During the method development, different chromatographic conditions were tested, considering the chemical structure and physicochemical properties of ritlecitinib. It is a pyrrolopyrimidine that contains three rings, three rotatable bonds, and six heteroatoms (one oxygen and five nitrogen atoms). Four of these are hydrogen acceptors, while two are hydrogen donors. Ritlecitinib is a white to off-white solid that is insoluble in water. Based on data calculated by the ALOGPS v2.1 program, its water solubility is 0.46 mg/mL. It is moderately lipophilic, with predicted log *P* data in the range of 1.27 (ACD/Labs v9.0 program) to 1.95 (Mcule v1.0 program). Ritlecitinib is a multiprotic compound with dissociation constant (p*K*_a_) values ranging from 6.6 (strongest basic) to 13.6 (strongest acidic).

This part of the study aimed to develop a chromatographic method that would allow for fast separation of ritlecitinib and its degradation products, as well as the quantitation of ritlecitinib in stressed samples. As a result, Core-Shell technology was employed as the stationary phase to achieve higher chromatographic efficiencies at substantially lower system backpressures. Furthermore, chromatographic columns packed with smaller particle size (sub-3 µm) were chosen to explore the benefits offered by these columns. Finally, the C18 column showed higher hydrophobic retention suitable for ritlecitinib and its non-polar degradation products in comparison to the C8 column. The reversed-phase C18 columns were also used by Bauman and co-workers [10] for ritlecitinib metabolite profiling, as well as by Salva and Galla [11] for the determination of ritlecitinib in rat plasma samples.

After the selection of the stationary phase, the mobile phase composition was optimized to achieve the required retention and selectivity of the chromatographic procedure. The mobile phase modifiers led to different eluent viscosity and UV cut-offs, and these factors were considered during the design of eluent systems as well. Since the volatility and the solvent’s ability to donate a proton are important in the electrospray ionization (ESI) technique, they were also scrutinized when designing eluent systems. Experimental trials revealed that using acetonitrile, compared to methanol and ethanol, resulted in improved peak shapes for ritlecitinib and its degradation products. Different mobile phase modifiers covering acidic to neutral pH were screened. Using lower pH values, solvents offered better separation and enhanced peak resolution. We observed that the detection sensitivity of ritlecitinib and its degradation products was improved using formic acid (p*K*_a_ 3.75) as an additive of the mobile phase for UHPLC-DAD-MS/MS analysis. Overall, these findings follow analytical methods reported by Bauman and co-workers [10] as well as Salva and Galla [11].

By analyzing the stressed samples, it became evident that fewer polar degradation products had suitable retention factor values with a lower percentage of aqueous mobile phase, while polar degradation products had appropriate retention factor values with a lower percentage of organic mobile phase. Therefore, the use of a gradient system for increasing eluting power was necessary.

Ritlecitinib showed good absorption between 200 and 300 nm; however, the wavelength of 280 nm was selected for analysis to achieve superior sensitivity and peak shape.

By applying optimized chromatographic conditions, parameters defining the system’s suitability were determined. The system suitability parameters for the proposed method were established at the target concentration of ritlecitinib (10 µg/mL). The proposed analytical method meets ICH and USP standards for system appropriateness. The observed retention time of ritlecitinib was 3.77 min with a method total run time of 13 min, making the method both time- and cost-effective. According to Table 1, the relative standard deviation (RSD) values did not surpass the recommended value of 2%. Compliance with these factors is crucial in verifying the system’s appropriateness for its intended purpose.

### 2.2. Method Validation

Using guidelines (Validation of analytical procedures, ICH Q2(R2)), various parameters for the developed stability-indicating method were validated, including linearity, limit of detection (LOD), limit of quantitation (LOQ), precision, accuracy, and standard solution stability [12].

The ritlecitinib solutions, ranging from 2 to 100 µg/mL, were utilized to create the linearity curve. Table 2 contains the results of the linear regression study. With a correlation coefficient (*r*) value of 0.999, the results demonstrate a significant correlation between observed responses and ritlecitinib concentrations. In addition, the accuracy of the back-calculated concentrations of each calibration standard was evaluated, revealing relative errors within ±2.24% of the theoretical concentration at all investigated levels. Furthermore, residual analysis was conducted to assess the assumptions of linearity and homoscedasticity (Figure S1). The visual evaluation of the pattern of the residual versus fits plot showed that the residuals appeared to be randomly dispersed around the zero, with no recognizable patterns in the points, revealing that the proposed linear model approximates the data points well without favoring certain inputs.

The LOD and LOQ values were determined experimentally, the method based on signal-to-noise (S/N) ratio was applied. The LOD (S/N = 3) and LOQ (S/N = 10) values were found to be 0.04 and 0.14 µg/mL, indicating the sensitivity of the method (Table 2).

Method precision was determined by repeatability (intraday), where the analysis of samples at the same concentrations was evaluated on the same day at different time intervals, and by intermediate precision (interday), where the samples were evaluated by comparing the assays on three separate days. Each day six sample solutions (10 µg/mL) were prepared and analyzed; the assay value was expressed in terms of RSD values and the box-and-whisker plot (Table 2, Figure S2). Low values of RSDs, ranging from 0.12 to 0.15%, demonstrate acceptable method precision.

Method accuracy was calculated using the regression equation based on percentage recoveries at three concentration levels (low-level 2 µg/mL, medium-level 10 µg/mL, and high-level 100 µg/mL) crossing the linearity curve. All solutions prepared and analyzed in triplicate were experimentally recovered (99.9–100.3%) with low values of RSD (lower than 0.7%) during analysis (Table 2).

The experimental results demonstrate the high stability of the standard solution. Despite being stored for 8 h at room temperature, for 3 days in a laboratory refrigerator (4 °C), and for 7 days in a laboratory freezer (−20 °C), the stability testing solutions showed excellent agreement with the freshly prepared samples. All stability tests showed no significant difference in ritlecitinib concentrations, with recoveries ranging from 99.2 to 99.7% (Table 2).

### 2.3. Forced Degradation Study

**Figure 2:**
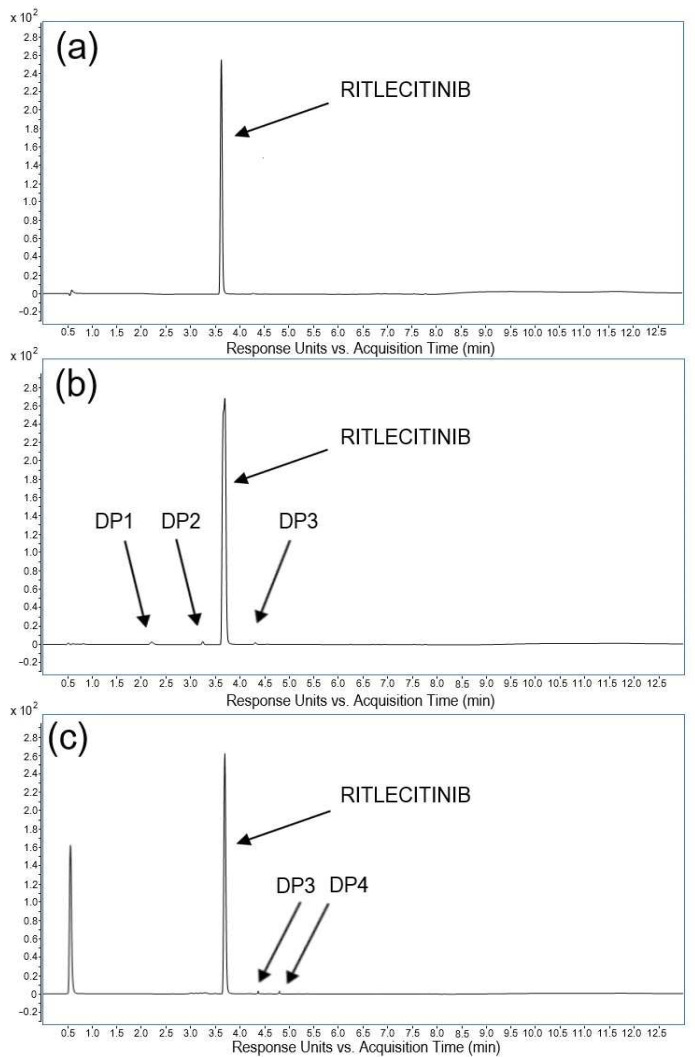
Representative LC chromatograms of ritlecitinib samples (100 µg/mL): (**a**) control, (**b**) basic, and (**c**) oxidative degradation.

Intentional degradation was attempted at stress conditions of acidic (using 0.01–1 M HCl), basic (using 0.01–1 M NaOH), oxidative (using 3–33% H_2_O_2_), photolytic (daylight degradation), and thermal degradation (heated at 40 and 70 °C) to evaluate the ability of the proposed analytical method to separate ritlecitinib and its degradation products [13,14]. A critical aspect of designing well-structured forced degradation experiments was defining the severity and exposure time for each condition, along with providing meaningful endpoints. The two endpoints, the degradation of the drug molecule up to 10–20% or an exposure time of no more than 10 days, were selected to ensure thorough coverage of pharmaceutically-relevant degradation. The analysis of all stress samples revealed that the retention times for ritlecitinib remained unaltered compared to the control solution (*t*_R(average)_ = 3.74 min; RSD = 1.1%). The peak purity of ritlecitinib in all stress samples was above 999.1, with the resolution between the peaks of ritlecitinib and degradation products higher than 6.5. Thus, the concentration of ritlecitinib in stress samples was reliably quantified, and the degradation products were separated, indicating the high specificity of the proposed method as well as its stability-indicating power. The chromatograms depicting the degradation behavior of the ritlecitinib drug substance under different stress conditions are shown in Figure 2.

The results for the acidic, basic, oxidative, thermal, and photolytic degradation of ritlecitinib are presented in Table 3. Four novel degradation products, which have not been previously reported, were identified under the applied stress conditions. As per the elution pattern, the degradation products were denoted as DP1 to DP4 (Table 4).

Over 72 h, ritlecitinib significantly degraded through basic hydrolysis at room temperature. This stress condition resulted in a degradation of 12.6% of ritlecitinib with the appearance of three degradation peaks. Relative retention times (RRTs) of DP1 and DP2 were lower than 1.0, indicating lower hydrophobicity of these degradation products, while the RRT value of DP3 was higher than 1.0, indicating its higher hydrophobicity compared to ritlecitinib.

The degradation of ritlecitinib under oxidative stress, using 33% hydrogen peroxide solution, occurred rapidly, achieving a degradation of 9.5% of ritlecitinib within 48 h. The chromatogram obtained from analyzing oxidative stress illustrates a distinct separation between ritlecitinib and two more hydrophobic degradation products, namely DP3 and DP4.

The acidic degradation study was carried out in 1 M HCl for 10 days under reflux conditions, at room temperature and 70 °C. It was revealed that ritlecitinib is stable to acidic hydrolysis, with a loss of ritlecitinib of less than 1%. For thermal degradation, the ritlecitinib standard solution was kept at 40 and 70 °C for 10 days, but no appreciable degradation occurred under these conditions (less than 1%). The photolytic degradation of ritlecitinib resulted in a loss of analyte of 1% without the formation of a degradant peak.

Our results underscored ritlecitinib’s vulnerability to basic and oxidizing conditions. It was noticed that it was not sensitive to acidic conditions, temperature, and light. These data provide invaluable insights into the stability profile of ritlecitinib, guiding the optimization of synthesis conditions, formulation development, package development of pharmaceutical products, and the development of storage protocols. Moreover, by addressing the stability-related challenges early in the development process, the production of high-quality ritlecitinib formulations with innovative excipients can be ensured.

After testing for accelerated degradation, a kinetic study of basic and oxidative degradation of ritlecitinib was performed. For this purpose, samples were collected at predefined time intervals (at least ten data points were collected before reaching 50% drug degradation). Various kinetic models were used and compared to examine the rate of the basic and oxidative degradation processes. The reaction order was determined by plotting the drug concentration (zero-order), the logarithm of drug concentration (first-order), and the reciprocal of drug concentration (second-order) versus time (all equations are given in Table S1). The regression equations were computed, the correlation coefficients were calculated, and the reaction order was identified based on the best-fitting model.

Based on the correlation coefficients obtained for zero-order (*r* = 0.983), first-order (*r* = 0.989), and second-order model fits (*r* = 0.994) in the basic degradation kinetic study, it was concluded that the basic degradation of ritlecitinib follows second-order kinetics. Accordingly, basic degradation of ritlecitinib mainly depends on the concentration of the drug substance and stressor.

The correlation coefficient for the zero-order kinetics model in the oxidative degradation kinetic study was 0.999. It was higher than the coefficients derived from first-order (*r* = 0.997) and second-order model fits (*r* = 0.993). This suggests that the oxidative degradation process followed the zero-order kinetics model, indicating that the oxidative degradation of ritlecitinib does not depend on the concentration of the drug and stressor. It is by now generally accepted that zero-order reactions primarily include rearrangement or radical-mediated cleavage of chemical bonds under oxidative stress conditions [15].

Rate constant, half-life (*t*_50_), and shelf-life (*t*_90_) are the primary parameters measured in degradation kinetics, presented in Table 5. Both *t*_50_ and *t*_90_ values were found to be lower for oxidative degradation than basic hydrolysis.

### 2.4. Mass Spectrometry of Ritlectinib and Its Degradation Products

**Figure 3:**
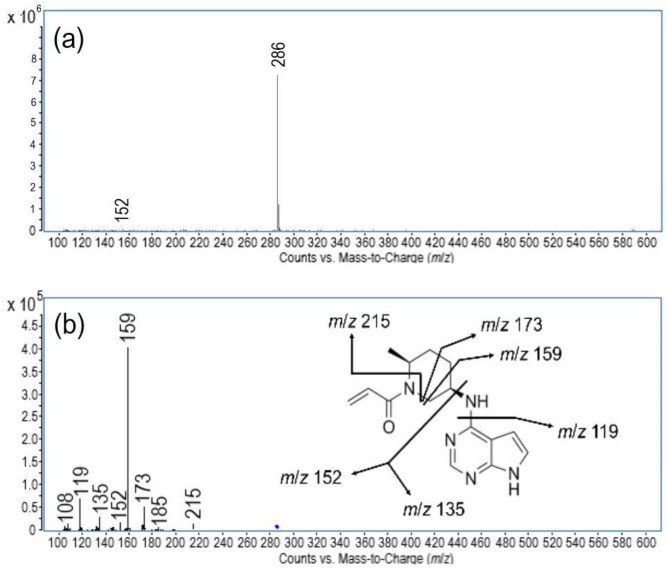
Mass spectra data of ritlecitinib. (**a**) Full scan ESI-MS spectrum, showing the protonated molecular ion [M+H]^+^ at *m*/*z* 286. (**b**) Product ion ESI-MS/MS spectrum of the protonated molecular ion [M+H]^+^ (*m*/*z* 286), collision energy of 20 V (insert: proposed fragmentation pattern).

The mass spectrometric experiments were conducted for the mass identification of newly generated degradation products (DP1–DP4). Monitoring was carried out following a detailed analysis of the mass spectrum of ritlecitinib and its fragmentation products, enabling the identification of the characteristic fragmentation processes involved (Figure 3). Under positive ESI-MS conditions, the prominent molecular peak [M + H]^+^ at *m*/*z* 286 was observed, confirming that the molecular formula for ritlecitinib is C_15_H_19_N_5_O. Thereafter, MS/MS experiments were conducted to acquire a thorough understanding of its fragmentation behavior, facilitating the establishment of a defined fragmentation pattern for ritlecitinib and its degradation products. The fragmentation pattern observed for ritlecitinib is shown in Figure 3. A total of six fragments were distinguished. The first fragment, having *m*/*z* 215, was obtained due to the neutral loss of the prop-2-enamide. Further cleavage of the piperidine ring resulted in the formation of the fragment at *m*/*z* 173. The loss of the methyl group gave rise to the prominent fragment having *m*/*z* 159. The next fragments, with *m*/*z* 152 and 135, were observed due to cleavage around the central nitrogen. The fragment ion at *m*/*z* 119 was generated due to the removal of the amino group from the fragment at *m*/*z* 135, forming 7H-pyrrolo [2,3-d]pyrimidine. To our knowledge, only one report on the pharmacokinetics, metabolism, and clearance mechanism of ritlecitinib, including its mass spectrometric data, is available in the literature [10]. Our proposed fragmentation pattern of ritlecitinib bears a close resemblance to the previously published one. However, due to the careful optimization of the mass spectrometry parameters, our procedure revealed three new fragments.

**Figure 4:**
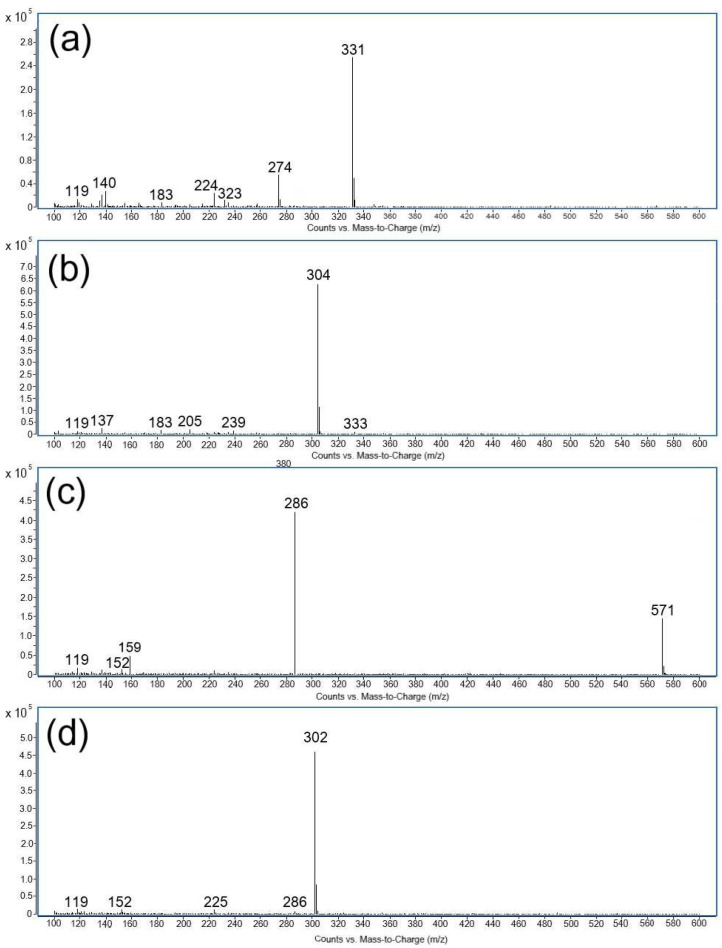
Positive single scan ESI-MS spectra of degradation products: (**a**) Degradation Product 1—DP1 (basic hydrolysis), (**b**) Degradation Product 2—DP2 (basic hydrolysis), (**c**) Degradation Product 3—DP3 (basic hydrolysis/oxidative stress), and (**d**) Degradation Product 4—DP4 (oxidative stress).

The formation of degradation products was evaluated based on the mass fragmentation pattern of ritlecitinib as well as its degradation products, identifying the underlying chemical transformations. Furthermore, this was complemented by the EPFL Mass Spectrometry online tool, chemical knowledge, and the conditions of impurity formation. The mass spectra of all four degradation products (DP1-DP4) are shown in Figure 4. The retention times, relative retention times (RRTs), main peaks, along with major fragments of all the degradation products, are summarized in Table 4.

DP1 (base peak at *m*/*z* 331) and DP2 (base peak at *m*/*z* 304), derived from the basic hydrolysis of ritlecitinib, showed RRT values lower than 1. Both degradation products generated a simple MS spectrum with the formation of one base peak and several minor fragment ions. In the case of DP2, the experimental *m*/*z* value of the base peak was 304, which is 18 Da higher than the molecular ion of ritlecitinib [M  +  H]^+^. It is presumed that this polar product is generated under basic conditions due to the 1,4 addition of a hydroxyl group across the alkene bond of the α, β-unsaturated carbonyl present in the acrylamide moiety of ritlecitinib.

DP3 is produced under both stress conditions, basic hydrolysis and oxidative stress. The most important peak of DP3 was found to be at *m*/*z* 571, as shown in Figure 4 and Table 4. Due to its apolar nature, it elutes after ritlecitinib with an RRT of 1.16. Three ions were also observed for DP3, having *m*/*z* 286, 159, 152, and 119. Ritlecitinib’s structure contains an acrylamide moiety, which is prone to the formation of dimers featuring either C-C or C-N linked chains, with the latter arising from Michael addition. Based on the aforementioned data on the MS spectrum of DP3 and its apolar nature, a possible structure of DP3 could be proposed as the ritlecitinib dimer. Recent work of Chum and co-workers [16] highlighted the importance of determining ritlecitinib dimers, as this impurity may affect the crystallization of the drug.

The second apolar degradation product of oxidative stress (DP4) was observed at the highest retention time (RRT = 1.27). In the MS spectrum, the base peak of DP4 was found to be at *m*/*z* 302 in positive mode, indicating a molecular weight of 301 Da, which is 16 Da higher than the molecular weight of ritlecitinib. As described in the literature, hydrogen peroxide can react with tertiary amines, which are susceptible to electron transfer oxidation, producing N-oxides [17]. The difference of 16 Da corresponds to oxygen and may be related to the formation of an N-oxide in the piperidine ring of ritlecitinib. Other peaks obtained in the MS spectrum of DP4 showed three characteristic peaks at *m*/*z* 286, 152, and 119. The formation of the peak at *m*/*z* 286 could be attributed to the loss of an oxygen atom [M + H − O]^+^. This fragmentation pattern has already been described for N-oxides [18]. Two other peaks at *m*/*z* 152 and 119 were also observed in the MS spectrum of ritlecitinib.

### 2.5. Assessment of Sustainability of Forced Degradation Procedure

We used seven green metric tools to evaluate the green performance of the developed forced degradation procedure and its subsequent impact on the environment.

The Analytical Method Greenness Score (AMGS) tool was used in the greenness assessment because it is a composite of several metrics, including the operator’s safety and procedure’s environmental impact [19]. The assigned greenness score of the forced degradation procedure of ritlecitinib, using the developed UHPLC/DAD/MS/MS method, was 711.85. Based on the calculated data, the highest contribution to the greenness score came from instrument energy (436.25, or 61.3% of overall score), followed by solvent influence (182.05, or 25.6% of overall score) and environment, health, and safety (99.55, or 13.1% of overall score). The scoring also includes a color-coding system that indicates the contribution of each category to the overall score. A green color highlights the green parameters of the method, while yellow and red colors indicate areas where the method can be improved. For the ritlecitinib forced degradation study, the category of instrument energy contributed over 50% of the overall AMGS score and is thus indicated in red. According to the AMGS score, the weakest point of our method is the energy consumption by the LC/MS technique; therefore, we attempted to reduce the instrument energy by shortening the method run time as much as possible.

The Analytical Eco-Scale [20] assigns a score out of 100, and our procedure accrued a total of 16 penalty points. Eleven penalty points were assigned due to the reagents used for the promotion of ritlecitinib degradation, two penalty points due to energy consumption of the LC/MS technique, and three points due to waste generation. Nonetheless, a score of 84 demonstrates the procedure’s strong ecological sustainability and environmental friendliness.

**Figure 5:**
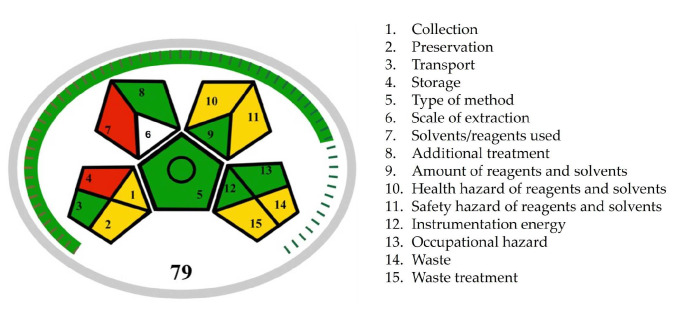
The MoGAPI score of ritlecitinib forced degradation protocol.

Płotka-Wasylka and co-workers [21,22] developed complementary metric tools Green Analytical Procedure Index (GAPI), along with its recent versions Modified Green Analytical Procedure Index (MoGAPI), and Complementary Green Analytical Procedure Index (ComplexGAPI). MoGAPI evaluated the environmental hazards of our procedure using a central pentagon surrounded by four additional pentagons mirroring each of its sides, excluding the bottom. Each pentagon assessed specific aspects of the methodology using three colors (green, yellow, and red), where green represents the most eco-friendly method aspects and red indicates non-ecofriendly method aspects (Figure 5). According to the MoGAPI pictogram, the ritlecitinib forced degradation study showed six green, six yellow, and two red areas. The yellow areas (fields 10, 11, 14, and 15) are related to the usage of organic solvents and formic acid as mobile phase modifiers, leading to chemical waste generation. The red areas (fields 4 and 7) are attributed to the preparation of forced degraded samples, particularly the use of stressors and high temperatures. We need to be aware that method development and validation, as well as the evaluation of degradation kinetics for ritlecitinib, included numerous sample analyses that needed to be performed. Furthermore, the use of stress conditions in forced degradation studies is inevitable. Therefore, during the method development, we prioritized the minimization of sample size and method run time and, alongside that, the minimization of waste production. The MoGAPI tool also offered an overall assessment of the method’s greenness by calculating a total score. The total score of 79 for the ritlecitinib forced degradation study appeared on the chart, and the green color of the scale around the pentagrams indicated a high overall greenness score.

**Figure 6:**
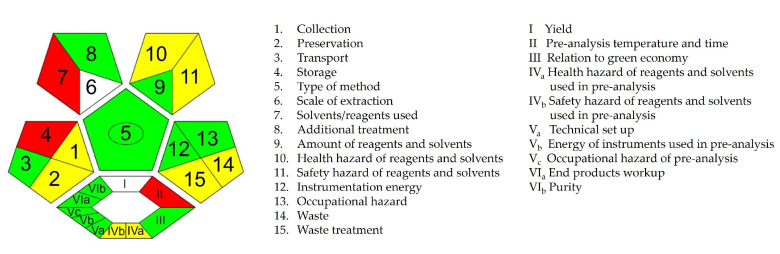
The ComplexGAPI score of ritlecitinib forced degradation protocol.

ComplexGAPI expands the pictogram by adding a hexagonal field at the bottom to represent the environmental impact of pre-analysis processes [22]. The ComplexGAPI pictogram indicated six green areas, two yellow areas, and one red area of the hexagonal grid (Figure 6). Again, the red area (field II) is attributed to the use of a high temperature for the thermal stress study, while the two yellow areas (fields IV_a_ and IV_b_) are attributed to the use of acids, bases, and oxidants for the induction of chemical stress, although low amounts of these reagents were used.

**Figure 7:**
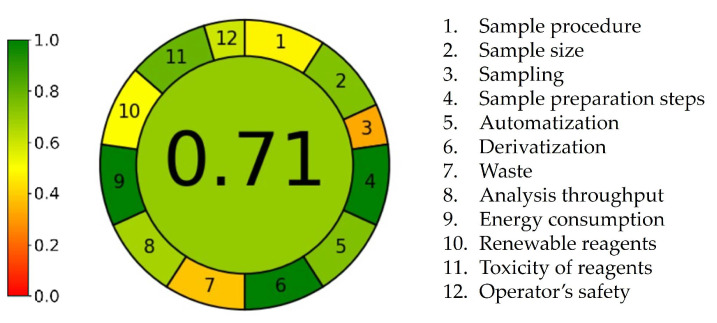
The AGREE score of ritlecitinib forced degradation protocol.

The Analytical GREEnness Metric Approach and Software (AGREE) v1.0 tool is based on twelve consecutive assessment steps that correspond to the ten principles of green analytical chemistry [23]. The result is presented as a round green pictogram with a circle in the center that shows an overall score of 0.71, indicating that the developed method complies with the principles of green analytical chemistry, despite several limitations (Figure 7). The segments display scores in the range of 0.33–1.00. A score of 1, indicating high greenness and depicted by the deepest shade of green, is assigned for three criteria (criteria 4, 6, and 9). Scores in the range of 0.60–0.80, with the color transitions gradually from dark green to light green, are assigned for five criteria (criteria 2 and 5 — score 0.75; criterion 8 — score 0.68; criterion 11 — score 0.80; and criterion 12 — score 0.60). As previously mentioned, one of the most critical points of our procedure is related to the usage of hazardous chemicals, making criteria 1 and 10 highlighted in yellow with scores of 0.48 and 0.50, respectively. Each sample analysis generates approximately 10 mL of waste, placing criterion 7 at the bottom of the scale (score 0.39, highlighted in orange). The main limitation regarding greenness is associated with the sampling procedure. Since in situ sampling cannot be applied in a forced degradation study, criterion 3 is scored with 0.33 and highlighted in orange. Despite these constraints, the overall methodology was considered environmentally friendly, as indicated by the high overall score, eight out of twelve green areas, and the absence of red areas in the AGREE pictogram.

**Figure 8:**
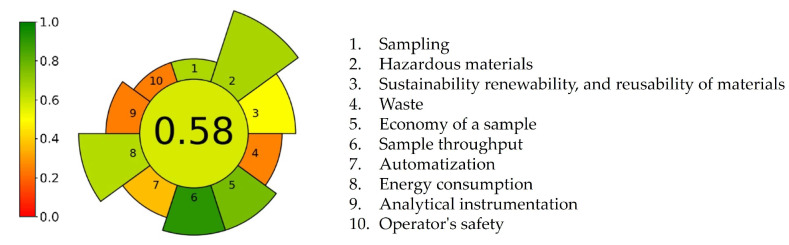
The AGREEprep score of ritlecitinib forced degradation protocol.

Sample preparation in the forced degradation study has been identified as one of the most critical steps from the perspective of green analytical chemistry [8,9], primarily due to the typical requirements for reagents and energy consumption. Thus, we used the Analytical GREEnness Metric for Sample Preparation (AGREEprep) tool, which emphasizes sample preparation. It predicted, as well as identified, aspects of the developed forced degradation study that could be improved for greening the critical step of sample preparation. The key strength of the AGREEprep tool lies in its comprehensive coverage of all ten green sample preparation principles, offering a detailed evaluation of each aspect through a diverse range of colors [24]. Figure 8 depicts a round AGREEprep pictogram with a circle in the middle that shows an overall score of 0.58, colored in light green. Ten trapezoid bars correspond to the ten criteria. Five out of ten criteria are marked with various shades of green, with scores between 0.64 and 0.92. These green points were obtained due to high sample preparation throughput, low sample size, and moderate consumption of hazardous reagents. The third principle of green sample preparation promotes the use of reusable, renewable, and sustainable resources. Since we utilized recycled materials and reusable laboratory glassware whenever possible, the corresponding bar is highlighted in yellow with a score of 0.5. Four bars, representing the waste generation (criterion 4, score 0.26), automatization of procedure (criterion 7, score 0.38), analytical technique used for sample analysis (criterion 9, score 0.25), and operator safety (criterion 10, score 0.25), are shown in orange, denoting the weakest points of the developed procedure.

**Figure 9:**
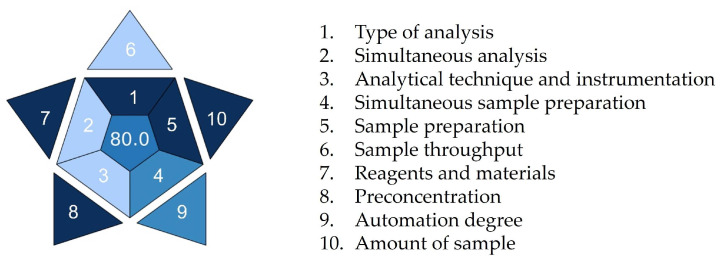
The BAGI score of ritlecitinib forced degradation protocol.

In recent years, the idea of white analytical chemistry has also been promoted as an extension and complement to green analytical chemistry. It combines the ecological, analytical, and practical perspectives of an analytical method [25]. To obtain a more comprehensive approach to the sustainability of the proposed procedure, the Blue Applicability Grade Index (BAGI) approach was applied [26]. Figure 9 shows an asteroid pictogram demonstrating the practicability of our procedure. A BAGI score of 80 was assigned to the procedure, demonstrating its superiority in terms of practicality and applicability. The shades of blue assigned to the individual pictogram fields indicate the weak and strong points of the analytical procedure. The biggest advantages of the proposed procedure are undemanding sample preparation, low sample quantity, and usage of commonly commercially available reagents (criteria 1, 5, 7, 8, and 10 highlighted in dark blue for high compliance). Semi-automated devices were used for sample preparation, making criteria 4 and 9 highlighted in blue for medium compliance. On the other hand, due to limited sample analysis capacity per hour, concurrent analyte determination capacity, and instrumentation requirements, criteria 2, 3, and 6 are highlighted in light blue for low compliance. It is encouraging that none of the procedure’s aspects were highlighted in white, demonstrating no compliance.

## 3. Materials and Methods

### 3.1. Chemicals

The ritlecitinib standard substance (assigned purity 99.98%) was purchased from MedChemExpress (Monmouth Junction, NJ, USA). Methanol, acetonitrile, and formic acid from Merck (Darmstadt, Germany) were of LC-MS grade. Sodium hydroxide pellets (97.0%, ACS reagent) and hydrogen peroxide solution (35% (*w*/*w*) in H_2_O) were acquired from Sigma-Aldrich (St. Louis, MO, USA), while hydrochloric acid (37%, p.a.) was purchased from Carlo Erba (Val-de-Reuil, France). Ultra-pure water was procured using the WaterPro water system (a resistivity of 18.2 mΩ·cm at 25 °C) Labconco (Kansas City, MI, USA).

### 3.2. Preparation of Stock and Standard Solutions

The stock solution of ritlecitinib (1 mg/mL) was prepared by dissolving an appropriate amount of the standard substance in methanol, followed by 15 min of sonication. Using a mixture of methanol and ultra-pure water (50:50, *v*/*v*), necessary aliquots from the stock solution were diluted to create the serial solutions in the required range of 2–100 µg/mL. Until they were used, the solutions were kept at 4 °C, protected from light.

### 3.3. Instruments and Analytical Conditions

The analytical equipment used comprised a liquid chromatograph Agilent 1260 series (auto-injector, binary pump, vacuum degasser, thermostat, and diode array detector) and an Agilent Ultivo triple quadrupole mass spectrometer with an ESI, connected to a computer loaded with OpenLab ChemStation v2.8 and MassHunter software v11.0 for equipment control, data acquisition, and processing.

Optimum separation of all analytes was achieved on a Kinetex EVO C18 Core-Shell analytical column (3.0 mm × 100 mm with 2.6 µm particle size) by Phenomenex (Torrance, CA, USA), with an operating temperature of 40.0 ± 0.1 °C, by employing ultra-pure water as mobile phase A, while acetonitrile served as mobile phase B; both were acidified with formic acid (0.1%) in gradient elution mode (0–4 min: 0–25% B; 4–6 min: 25–80% B; 6–13 min: 80–0%). A flow of 0.8 mL/min was utilized for the mobile phase. During a chromatographic run, the absorbance of the analytes was collected in the spectral range of 200 to 400 nm, while the wavelength of ritlecitinib assessment was determined to be 280 nm with a slit of 4 nm. The injection volume was 5 µL.

The MS settings were optimized to maximize the signal. The results were acquired in the positive mode obtained from the ESI source, operating at a capillary voltage of 3.5 kV and a nebulizer pressure of 20 psi. The gas temperature was maintained at 350 °C with a gas flow rate of 11 L/min. Daily calibration was performed using an ESI-L tuning mix (Agilent Technologies, Santa Clara, CA, USA). Mass spectra were recorded in full-scan mode over the *m*/*z* range of 100 to 600. The ritlecitinib fragmentation investigation employed a highly pure N_2_ (>99.9995) as the collision gas, with a collision energy of 20 V and a fragmentor voltage of 110 V.

### 3.4. Analytical Method Validation

Using ICH guidelines (Validation of analytical procedures, ICH Q2(R2)), a variety of parameters for the newly developed method for the assessment of ritlecitinib were validated [12].

### 3.5. Forced Degradation Study

The forced degradation study was conducted to prove the ability of the method to indicate stability. The testing was carried out as per ICH guidelines (Stability testing of new drug substances and drug products, Q1A(R2) and Photostability Testing of New Active Substances and Medicinal Products, Q1B) under different acid, base, oxidative, photolytic, and thermal conditions [13,14]. The forced degradation study was conducted either until a degradation level of 10–20% was achieved or for a maximum duration of 10 days. The samples were analyzed at zero time (without exposure) and at regular intervals after exposure to stress conditions. The optimal conditions found are described below. For acidic and basic degradation, the prepared stock solution was added to 1 M hydrochloric acid or 1 M sodium hydroxide and kept at ambient temperature in amber glass vials for 72 h for basic degradation and 10 days for acid degradation. For oxidative degradation, solutions were prepared in a hydrogen peroxide (33%) solution and kept at ambient temperature in amber screw cap vials for 48 h. Photodegradation of the sample solution was induced by a combination of UV and visible light for 10 days in transparent screw cap vials at room temperature. Finally, a thermal stress test was performed by storing the sample solution in amber screw cap vials within a thermostatically controlled incubator (ES-20/60, Biosan, Riga, Latvia) set at 70 °C for a period of up to 10 days. The stress samples were transferred to a volumetric flask and diluted with a mixture of methanol and ultra-pure water (50:50, *v*/*v*) to obtain a final concentration of 100 µg/mL. For every stress condition, the blank solution was subjected to stress in the same manner as the drug solution. The zero-time, blank, and stressed solutions were analyzed using the proposed UHPLC-DAD-MS/MS method. For kinetic study, samples were collected for up to 30 days until reaching 50% drug degradation. To define the kinetic model, equations presented in the Supplementary Materials (Table S1) were used. All calculations were obtained using Microsoft^®^ Excel v16.0 software.

### 3.6. Sustainability Assessment Methods

The sustainability of the forced degradation procedure was evaluated using the most widely applied greenness assessment tools such as AMGS, Analytical Eco-scale assessment method, MoGAPI, ComplexGAPI, AGREE, and AGREEprep. These approaches considered various aspects of the proposed analytical procedure to provide the green index of the forced degradation study [19,20,21,22,23,24]. Furthermore, BAGI, a new metric tool for evaluating the practicality of an analytical method, was applied [26]. The method input data are given in the Supplementary Materials (Tables S2–S8).

## 4. Conclusions

Following ICH recommendations, a UHPLC/DAD/MS/MS stability-indicating method for determining ritlecitinib and its degradation products was developed and validated. The stability of ritlecitinib was evaluated by exposing the substance to various stress conditions. The formation of degradation products was observed for the basic and oxidative stress conditions. A kinetic study was conducted, and the rate constant, half-life (*t*_50_), and shelf-life (*t*_90_) were determined. Oxidative degradation of ritlecitinib follows zero-order kinetics, while basic degradation follows second-order kinetics. The careful optimization of the mass spectrometry parameters led to the formation of six ritlecitinib fragments; among them, three were first reported in our study. The analysis showed four degradation products characterized for the first time. This study presents contributory data for future investigations on the ritlecitinib formulation development, determination of proper storage conditions, selection of container closure system, drug–excipient interactions anticipation, better stabilization of the drugs, and accurate prediction of shelf-life.

The eco-friendliness and applicability of the developed forced degradation procedure were evaluated using various metrics. As for the future perspective, we believe that the introduction of principles of green and white analytical chemistry into method development may contribute to an increase in the development of eco-friendly analytical procedures applicable in routine analysis.

## Figures and Tables

**Table 1:** Summarized system suitability data of the newly developed stability-indicating method (ritlecitinib concentration level 10 µg/mL; *n* = 6).

Parameter	Symbol	Value	RSD ^1^ (%)	Reference Values
Retention time (min)	*t*_R_	3.77	0.02	RSD < 2.0%
Peak area (mAU·s)	*A*	68.90	0.97	RSD < 2.0%
Peak purity	*P_p_*	999.8	0.20	>999.0
Peak capacity	*P_c_*	258	4.01	N/A ^2^
Symmetry	*A*_s_	1.00	0.78	0.8–1.2

^1^ RSD—Relative Standard Deviation; ^2^ N/A—Not Applicable.

**Table 2:** Summarized stability-indicating method validation data.

Validation Parameter	Value	Reference Values
Linearity		
Range (µg/mL)	2–100	N/A ^8^
Slope	18.31	N/A
Intercept	−6.59	N/A
SE ^1^ of the slope	0.020	N/A
SE of the intercept	0.939	N/A
Correlation coefficient (*r*)	0.999	0.990
Regression SS ^2^	3,439,121	N/A
Residual SS	30.06	N/A
Total SS	3,439,151	N/A
Sensitivity ^3^		
LOD (µg/mL)	0.04	N/A
LOQ (µg/mL)	0.14	N/A
Precision ^4^		
Repeatability (*n* = 6; RSD ^5^, %)	0.12	RSD < 2.0
Intermediate precision (*n* = 18; RSD, %)	0.15	RSD < 2.0
Accuracy ^6^		
Low (*n* = 3; mean recovery, %/RSD, %)	100.3/0.3	95–105/RSD < 2.0
Medium (*n* = 3; mean recovery, %/RSD, %)	99.9/0.7	95–105/RSD < 2.0
High (*n* = 3; mean recovery, %/RSD, %)	100.3/0.7	95–105/RSD < 2.0
Stability ^7^		
Benchtop stability (recovery, %)	99.7	95–105
Short-term stability (recovery, %)	99.5	95–105
Long-term stability (recovery, %)	99.2	95–105

^1^ SE—Standard Error; ^2^ SS—Sum of Squares; ^3^ The limit of Detection (LOD) and Limit of Quantitation (LOQ) were determined using signal-to-noise value 3 and 10, respectively; ^4^ The repeatability was assessed by analyzing standard solution (10 µg/mL) within the same day in six replicates, while the intermediate precision was assessed by analyzing standard solution on three consecutive days in six replicates; ^5^ RSD—Relative Standard Deviation; ^6^ The accuracy was performed by analyzing three concentration levels of standard solutions (low-level 2 µg/mL, medium-level 10 µg/mL, and high-level 100 µg/mL) in triplicate; ^7^ The standard solution (10 µg/mL) was stored at room temperature for a period of 8 h and analyzed thereafter to determine the benchtop stability of ritlecitinib. The short-term stability was carried out by storing the standard solution of ritlecitinib (10 µg/mL) at 4 °C for 72 h. For the long-term stability, the standard solution of ritlecitinib (10 µg/mL) was stored at −20 °C for 7 days; ^8^ N/A—Not Applicable.

**Table 3:** Summarized forced degradation study data.

Stress Type	Stress Condition	Exposed Conditions	Duration	Degradation of Ritlecitinib (%)	Ritlecitinib Retention Time (*t*_R_, min, Average ± RSD ^1^, *n* = 6)	Ritlecitinib Peak Symmetry Index (Average ± RSD, *n* = 6)	Remarks
Acidic hydrolysis	1 M HCl	70 °C	10 days	-	3.74 ± 0.02	0.911 ± 0.78	No degradation was observed
Basic hydrolysis	1 M NaOH	RT ^2^	72 h	12.6	3.79 ± 0.02	0.935 ± 0.45	Three degradation peaks observed: DP1, DP2, and DP3
Oxidation	33% H_2_O_2_	RT	48 h	9.5	3.76 ± 0.02	0.966 ± 0.07	Two degradation peaks observed: DP3 and DP4
Thermal	Elevated temperature	70 °C	10 days	-	3.73 ± 0.23	0.911 ± 0.70	No degradation was observed
Photodegradation	Daylight	RT	10 days	-	3.76 ± 0.77	0.918 ± 0.61	No degradation was observed

^1^ RSD—Relative Standard Deviation; ^2^ RT—Room Temperature.

**Table 4:** UHPLC-ESI-QQQ data of ritlecitinib and its degradation products.

Compound/Degradation Product	Stress Conditions	Retention Time (*t*_R_, min)	Relative Retention Time (RRT)	Retention Factor	Base/Main Peak (*m*/*z*)	List of Peaks (*m*/*z*)
Ritlecitinib	-	3.77	-	5.18	286	286, 215, 185, 173, 159, 152, 135, 119, 108
Degradation product 1 (DP1)	Basic hydrolysis	2.24	0.59	2.67	331	331, 274, 232, 224, 183, 140, 119
Degradation product 2 (DP2)	Basic hydrolysis	3.28	0.87	4.38	304	333, 304, 205, 183, 137, 119
Degradation product 3 (DP3)	Basic hydrolysis/Oxidative	4.37	1.16	6.16	571	571, 286, 159, 152, 119
Degradation product 4 (DP4)	Oxidative	4.79	1.27	6.85	302	302, 286, 225, 152, 119

**Table 5:** Degradation kinetics data for basic hydrolysis and oxidative degradation.

Degradation Condition	Duration of Kinetic Study (Days)	Order of Kinetic Degradation	Reaction Rate Constant (*k,* µgmL^−1^h^−1^)	Half-Life (*t*_50_, h)	Shelf-Life (*t*_90_, h)
Basic hydrolysis, 1 M NaOH, RT ^1^	30	Second-order kinetics	0.000016	662.5	66.3
Oxidative degradation, 33% H_2_O_2_, RT	12	Zero-order kinetics	0.181	266.8	53.4

^1^ RT—Room Temperature.

## Data Availability

The original contributions presented in this study are included in the article/Supplementary Materials, further inquiries can be directed to the corresponding authors.

## Supplementary Materials

The following supporting information can be downloaded at: https://www.mdpi.com/article/10.3390/ph18010124/s1, Figure S1: Residual plot of calibration curve of ritlecitinib; Figure S2: The box-and-whisker plot of intermediate precision data. The average value is indicated by a cross, the central line represents the median value, and the large box indicates the inter-quartile range (1st to 3rd quartiles). The upper and lower whiskers correspond to the highest and lowest values, respectively; Table S1: Orders of degradation reaction and equations for calculating kinetic parameters; Table S2: AMGS input data of ritlecitinib forced degradation procedure; Table S3: Analytical Eco-Scale input data of ritlecitinib forced degradation procedure; Table S4: MoGAPI input data of ritlecitinib forced degradation procedure; Table S5: ComplexGAPI input data of ritlecitinib forced degradation procedure; Table S6: AGREE input data of ritlecitinib forced degradation procedure; Table S7: AGREEprep input data of ritlecitinib forced degradation procedure; Table S8: BAGI input data of ritlecitinib forced degradation procedure.
