# Supplementary material for: The Stability-Indicating Ultra High-Performance Liquid Chromatography with Diode Array Detector and Tandem Mass Spectrometry Method Applied for the Forced Degradation Study of Ritlecitinib: An Appraisal of Green and Blue Metrics
Source: Pharmaceuticals (Basel). 2025 Jan 17;18(1):124. doi: 10.3390/ph18010124 (PMC11768339; doi:10.3390/ph18010124)
Supplement: Supplementary file 1 [file pharmaceuticals-18-00124-s001.zip › pharmaceuticals-3385763-supplementary.pdf]

Supplementary material

The stability-indicating UHPLC-DAD-MS/MS method applied for the forced degradation study of ritlecitinib: an appraisal of green and blue metrics

Jelena Kovačić, Daniela Amidžić Klarić, Nikša Turk, Željko Krznarić, Emma Riordan, Ana Mornar*

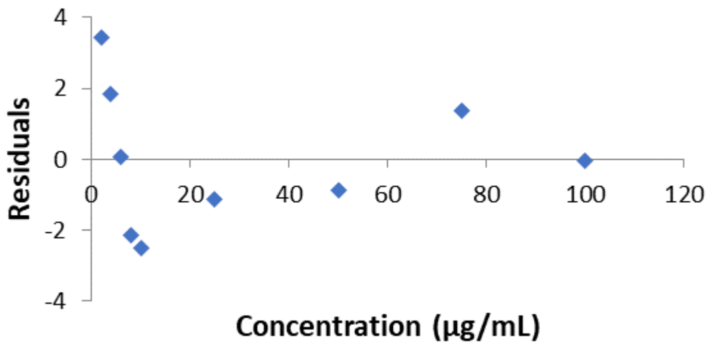

Figure S1. Residual plot of calibration curve of ritlecitinib.

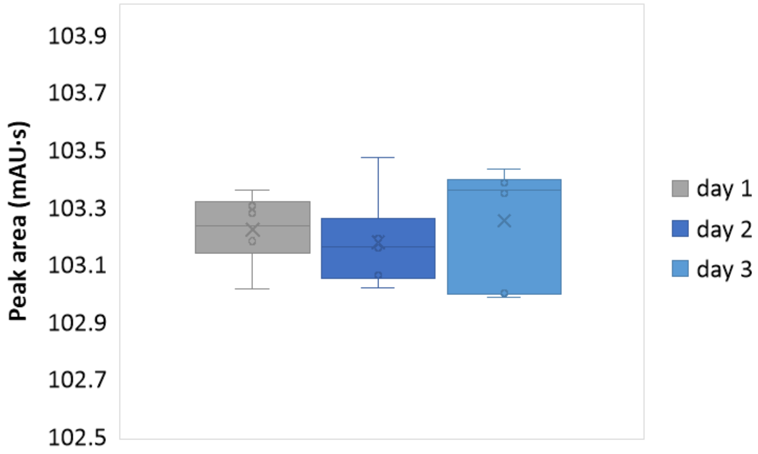

Figure S2. The box-and-whisker plot of intermediate precision data. The average value is indicated by a cross, the central line represents the median value, and the large box indicates the inter-quartile range (1st to 3rd quartiles). The upper and lower whiskers correspond to the highest and lowest values, respectively.

Table S1. Orders of degradation reaction and equations for calculating kinetic parameters.

Order of degradation reaction	Rate equation	Half-life equation	Shelf-life equation
Zero-order	R = $\frac{-d[A]}{dt} = k_0$	$t_{1/2}$ = $[A]_0$ / 2k	$t_{0.9}$ = 0.1$[A]_0$ / k_0
First-order	R = $\frac{-d[A]}{dt} = kA$	$t_{1/2}$ =ln(2) / k	$t_{0.9}$ = 0.105 / k_1
Second-order	d[A] = $\frac{-k[A]^2}{dt^2}$	$t_{1/2}$ =1 / k$[A]_0$	$t_{0.9}$ = (0.11 / k) $[A]_0$

Table S2. AMGS input data of ritlecitinib forced degradation procedure.

Criterion	Input data
Technique	LC-MS
Number of analytes of interest	5
Number of injections/runs for one full analysis	55
Flow rate (mL/min)	0.8
Run time (min/injection)	13
Gradient	0-4 min 0-25% B; 4-6 min 25-80% B; 6-13 min 80-0%
Mobile phase A	water (100%)
Mobile phase B	acetonitrile (100%)
Sample preparation volume (mL)	0.5
Number of samples	5
Sample preparation diluent	methanol : water (1:1, *v/v*)
Stock standard preparation volume (mL)	10
Number of stock standard	1
Stock standard diluent	methanol : water (1:1, *v/v*)
Working standard preparation volume (mL)	0.5
Number of standard solutions	42
Working standard diluent	methanol : water (1:1, *v/v*)
System suitability preparation volume (mL)	0.5
Number of system suitability solutions	1
System suitability diluent	methanol : water (1:1, *v/v*)
Sensitivity solution preparation volume (mL)	0.5
Number of sensitivity solutions	1
Sensitivity solution diluent	methanol : water (1:1, *v/v*)

Table S3. Analytical Eco-Scale input data of ritlecitinib forced degradation procedure.

Criterion	Input data
Penalty points due to reagents used	
acetonitrile	2
methanol	3
formic acid	1
sodium hydroxide	1
hydrochloric acid	2
hydrogen peroxide	2
Penalty points due to instruments used	
LC/MS	2
ultrasonic bath	0
ultrapure water system	0
magnetic mixer	0
vortex	0
thermostat	0
occupational hazard	0
waste	3

Table S4. MoGAPI input data of ritlecitinib forced degradation procedure.

Code	Criterion	Input data
Sample preparation		
C 1	Collection	On-line or at-line
C 2	Preservation	Chemical or physical
C 3	Transport	None
C 4	Storage	Under special conditions
C 5	Type of method	No sample preparation
C 6	Scale of extraction	Not applicable
C 7	Solvent/reagents used	Non-green solvents/reagents
C 8	Additional treatments	None
Reagents and solvents		
C 9	Amount	< 10 mL (< 10 g)
C 10	Health hazard	Moderately toxic; could cause temporary incapacitation, NFPA = 2 or 3
C 11	Safety hazard	Highest NFPA flammability or instability score = 2 or 3, or a special hazard is used.
Instrumentation		
C 12	Energy	≤ 0.1 kWh per sample
C 13	Occupational hazard	Hermetic sealing of analytical procedure
C 14	Waste	1-10 mL (1-10 g)
C 15	Waste treatment	Degradation
C 16	Quantification	Yes

Table S5. ComplexGAPI input data of ritlecitinib forced degradation procedure.

Code	Criterion	Input data
Sample preparation and analysis		
Sample preparation		
C 1	Collection	On-line or at-line
C 2	Preservation	Chemical or physical
C 3	Transport	None
C 4	Storage	Under special conditions
C 5	Type of method	No sample preparation
C 6	Scale of extraction	n.a.
C 7	Solvent/reagents used	Non-green solvents/reagents
C 8	Additional treatments	None
Reagents and solvents		
C 9	Amount	< 10 mL (< 10 g)
C 10	Health hazard	Moderately toxic; could cause temporary incapacitation, NFPA = 2 or 3
C 11	Safety hazard	Highest NFPA flammability or instability score = 2 or 3, or a special hazard is used.
Instrumentation		
C 12	Energy	≤ 0.1 kWh per sample
C 13	Occupational hazard	Hermetic sealing of analytical procedure
C 14	Waste	1-10 ml (1-10 g)
C 15	Waste treatment	Degradation
Method type		
O	Type of analysis	Qualitative and quantitative
Pre-analysis processes		
Yield and conditions		
C I	Yield	n.a.
C II	Temperature/time	Heating > 1 h, cooling < 0 °C
Relation to green economy		
C III	Number of rules met	5-6
Reagents and solvents		
C IV_a	Health hazard	Moderately toxic; could cause temporary incapacitation, NFPA = 2 or 3
C IV_b	Safety hazard	Highest NFPA flammability or instability score = 2 or 3, or a special hazard is used.
Instrumentation		
C V_a	Technical setup	Common setup
C V_b	Energy	≤ 0.1 kWh per sample
C V_c	Occupational hazard	Hermitization of analytical process
Workup and purification		
C VI_a	End product workup, purification	None or simple process
C VI_b	Purity	> 98%

Table S6. AGREE input data of ritlecitinib forced degradation procedure.

Code	Criterion	Input data
C 1	Sampling procedure	Off-line analysis (weight 2)
C 2	Amount of sample (g or mL)	0.5 (weight 2)
C 3	Positioning of the analytical advice	At-line (weight 1)
C 4	Sample preparation steps	3 or fewer (weight 2)
C 5	Integration and automatization	Semi-automatic; none or miniaturized (weight 2)
C 6	Derivatization agents	None (weight 2)
C 7	Amount of waste (g or mL)	10 (weight 2)
C 8	Sample throughput	Number of analytes determined in a single run: 5; Number of samples analysed per hour: 4 (weight 2)
C 9	Energy consumption	LC-MS; the power consumption of a single analysis: 0.04 (weight 2)
C 10	Reagents	Some reagents are bio-based (weight 2)
C 11	Toxic reagents and solvents	Yes; 0.1 mL (weight 2)
C 12	Threats	Highly flammable; highly oxidizable (weight 1)

Table S7. AGREEprep input data of ritlecitinib forced degradation procedure.

Code	Criterion	Input data
C 1	Sampling preparation and placement	On-line / In situ (weight 1)
C 2	Hazardous materials (g or mL)	0.1 (weight 5)
C 3	Sustainability, renewability, and reusability of materials	50-75% of reagents and materials are sustainable or renew, but can be used ONCE (weight 2)
C 4	Waste (g or mL)	10 (weight 2)
C 5	Size economy of the sample (g or mL)	0.5 (weight 3)
C 6	Sample throughput	50 (weight 3)
C 7	Integration and automatization	3 steps; semi-automated systems (weight 2)
C 8	Energy consumption (Wh)	40 (weight 4)
C 9	Post-sample preparation configuration for analysis	Liquid chromatography, gas chromatography with quadropole detection, etc. (weight 2)
C 10	Operator’s safety	3 hazards (weight 1)

Table S8. BAGI input data of ritlecitinib forced degradation procedure.

Code	Criterion	Input data
C 1	Type of analysis	Quantitative and confirmatory
C 2	Multi- or single-element analysis	Multi-element analysis for 2-5 compounds of the same chemical class
C 3	Analytical technique	Sophisticated instrumentation (LC-MS, GC-MS, ICP-MS, homemade interfaces, homemade automatic systems, etc.)
C 4	Simultaneous sample preparation	13-95
C 5	Sample preparation	Not required or on-site sample preparation if required
C 6	Samples per h	2-4
C 7	Reagents and materials	Common commercially available reagents (methanol, acetonitrile HNO_3, nitrogen or other common gases, etc.)
C 8	Preconcentration	No preconcentration required. Required sensitivity and / or legislation criteria are met directly.
C 9	Degree of automatization	Semi-automated with common devices (e.g. HPLC autosampler)
C 10	Amount of sample	< 100 µg (or mg) bioanalytical samples; < 10 mL (or g) food / environmental
